# Application of Liposomes in Treatment of Rheumatoid Arthritis: Quo Vadis

**DOI:** 10.1155/2014/978351

**Published:** 2014-02-04

**Authors:** Bhupinder Kapoor, Sachin Kumar Singh, Monica Gulati, Reena Gupta, Yogyata Vaidya

**Affiliations:** School of Pharmaceutical Sciences, Lovely Professional University, Phagwara, Punjab 144411, India

## Abstract

The most common treatments for rheumatoid arthritis include nonsteroidal anti-inflammatory drugs (NSAIDs), corticosteroids, disease modifying antirheumatic drugs (DMARDs), and some biological agents. However, none of the treatments available is able to achieve the ultimate goal of treatment, that is, drug-free remission. This limitation has shifted the focus of treatment to delivery strategies with an ability to deliver the drugs into the synovial cavity in the proper dosage while mitigating side effects to other tissues. A number of approaches like microemulsions, microspheres, liposomes, microballoons, cocrystals, nanoemulsions, dendrimers, microsponges, and so forth, have been used for intrasynovial delivery of these drugs. Amongst these, liposomes have proven to be very effective for retaining the drug in the synovial cavity by virtue of their size and chemical composition. The fast clearance of intra-synovially administered drugs can be overcome by use of liposomes leading to increased uptake of drugs by the target synovial cells, which in turn reduces the exposure of nontarget sites and eliminates most of the undesirable effects associated with therapy. This review focuses on the use of liposomes in treatment of rheumatoid arthritis and summarizes data relating to the liposome formulations of various drugs. It also discusses emerging trends of this promising technology.

## 1. Introduction

Rheumatoid arthritis (RA) is a systemic autoimmune inflammatory disease that affects the multiple joints of the body in a symmetric pattern [1, 2]. It is characterised by chronic inflammation of synovial membrane which often leads to destruction of articular cartilage, periarticular bone erosion, and permanent deformities. Classically, it causes synovitis in the metacarpophalangeal and proximal interphalangeal joints in a symmetrical manner. Clinically, it is manifested as warmth, swelling, tenderness with loss of motion, and grip strength in hands. RA commonly affects the feet, wrists, and knees, as well as cervical spine, shoulders, and hips [3]. At least 50% of patients with RA experience work disability within 10 years of onset of disease [4]. RA can also have systemic effects such as subcutaneous nodule development, pleural effusion, and pericarditis [5].

The prevalence of RA in general population has been estimated to be 0.8% and the incidence of RA in women is 3–5 times higher than in men [6, 7]. In India and China alone, about 19 million people are affected by RA [8]. Although it affects persons of all age groups, it is particularly prevalent in middle age population of 30–50 years. The mean life expectancy of patients suffering from RA has been reported to be reduced by 5–10 years; however, this also depends on severity of the disease [9].

**Figure 1:**
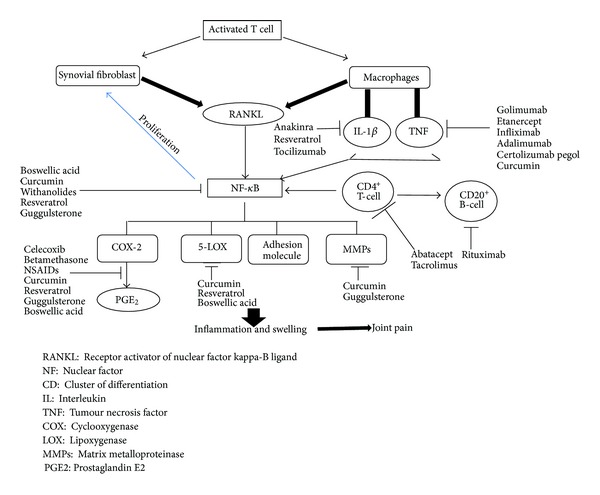
Etiology of rheumatoid arthritis and potential therapeutic agents and their sites of action.

The precise etiology of RA is not known, but it is evident that proinflammatory cytokines such as tumor necrosis factor-*α* (TNF-*α*), interleukin-1 (IL-1), interleukin-6 (IL-6), and transforming growth factor-*β* (TGF-*β*) play an important role in pathogenesis of disease [10]. These inflammatory cytokines are released by synovial macrophages, B cells, fibrocytes, synoviocytes, CD4^+^, and CD8^+^ T cells and can be detected in the synovium immunohistochemically [11]. In RA, the activated synoviocytes exhibit invasive growth into the joint cartilage and stimulate the differentiation and proliferation of osteoclasts which is responsible for bone erosion. The joint destruction is believed to be mediated mainly by cytokine-induced destructive enzymes, particularly members of metalloproteinase [12]. The activated synoviocytes are also responsible for progression of disease from arthritic joint to unaffected joints [9]. The etiology of RA is shown in Figure 1. The current review presents a detailed discussion about various agents used for the treatment of RA and the potential of novel drug delivery systems, particularly liposomes, to achieve successful delivery of these agents.

## 2. Potential Agents against Rheumatoid Arthritis

The diagnosis and early therapy of RA are very crucial, because, if untreated, up to 30% patients with newly diagnosed RA are unable to work within 3 years of diagnosis [7]. At present, there is no cure of RA and it is most commonly treated with a combination of nonsteroidal anti-inflammatory drugs (NSAIDs), corticosteroids, disease modifying antirheumatic drugs (DMARDs), and biological agents [13–16]. The treatment also involves the use of unconventional therapies such as enzymes like superoxide dismutase, antisense oligodeoxynucleotides, boron neutron capture therapy, and radioisotopes [17].

## 3. Nonsteroidal Anti-Inflammatory Drugs

Nonsteroidal anti-inflammatory drugs are commonly prescribed in the management of osteoarthritis, RA, and musculoskeletal pain. They only provide symptomatic relief and do not alter the course of the disease or prevent joint damage [5, 18, 19]. Mostly NSAIDs act by nonselective inhibition of cyclooxygenase (COX) enzyme which exists in two distinct isoforms, COX-1 and COX-2. Both these enzymes have nearly 60% amino acid homology, similar tertiary structure, and similar but nonidentical active sites [20]. COX enzyme catalyses the transformation of arachidonic acid into prostaglandins which are the mediators in the inflammatory process. Thus, inhibition of COX by NSAIDs leads to reduction in pain and inflammation [6]. COX-1-derived prostaglandins regulate many physiological processes such as protection of stomach lining from gastric acid erosion and vascular haemostasis. In contrast, COX-2 is principally an inducible enzyme which is highly expressed in inflammatory conditions. Therefore, selective inhibitors of COX-2 (Coxibs) are preferred over nonselective inhibitors [20]. The use of NSAIDs in RA is currently limited due to high risk of gastrointestinal complications. The gastrointestinal adverse effects range from minor discomfort to life-threatening peptic ulcers. The minor adverse effects include dyspepsia, heartburn, anorexia, abdominal pain, nausea, flatulence, or diarrhoea in 10% to 60% of patients. It has been reported that 15% to 35% of peptic ulcer complications are due to NSAIDs. NSAIDs and coxibs also cause renal and cardiovascular complications like acute kidney failure, hypertension, electrolyte abnormalities, myocardial infarction, and stroke [6, 18, 19, 21].

## 4. Glucocorticoids 

Glucocorticoids such as prednisone, methyl prednisone, hydrocortisone, triamcinolone, and dexamethasone are used to suppress the inflammation in RA and other autoimmune diseases [7]. They act by multiple mechanisms including inhibition of macrophage accumulation and reduction of capillary permeability [5]. Although they are most potent anti-inflammatory drugs and exhibit rapid onset of action, long term use of steroids is associated with severe side effects, including impaired wound healing, skin atrophy, osteoporosis, muscle atrophy, cataract, glaucoma, peptic ulcer, manifestation of latent diabetes, and ultimately premature mortality. These side effects can be minimised by using glucocorticoids at low dose particularly in patients unresponsive to NSAIDs and DMARDs or by administration of selective glucocorticoid receptor agonists that selectively target the immune and inflammatory pathways in order to reduce systemic toxicity or by intra-articular injection [5, 6].

## 5. Disease Modifying Anti-Rheumatic Drugs (DMARDs)

A number of disease modifying antirheumatic drugs (DMARDs) are available for treatment of RA. DMARDs can be further classified into traditional DMARDs comprising of a variety of small synthetic molecules and biological DMARDs produced by genetic engineering [6, 22]. Among DMARDs, methotrexate is the first choice of drug for the management of RA due to rapid onset, low cost, good response, and long-term safety [23]. Other traditional DMARDs used for management of RA include sulfasalazine, clodronate, hydroxychloroquine, and leflunomide. Some rarely used DMARDs include gold salts, D-penicillamine, azathioprine, cyclosporine, and tetracyclines [5, 14, 24]. However, the use of DMARDs is associated with side effects such as digestive organ dysfunction, liver dysfunction, kidney dysfunction, stomatitis, depilation, and myelosuppression [22, 25].

## 6. Biologics

In RA, the proinflammatory cytokines are overproduced in the joint cavity that induce joint destruction. In the recent years, certain biologics have been developed which inhibit the production of these cytokines [2]. The various biologics used for treatment of RA include tumour necrosis factor-*α* (TNF-*α*) antagonists, for example, etanercept, infliximab, adalimumab, and interleukin (IL)-1 receptor antagonist anakinra. A number of new biologics have been approved or are in the clinical development such as IL-6 inhibitor (tocilizumab), modified TNF-*α* antagonists (golimumab and certolizumab pegol), and monoclonal antibodies against various cytokines or targeting *β*-cells (ocrelizumab and ofatumumab). Biologics are not routinely prescribed for all the patients with RA due to cost factor ($16,000–$20,000 per year) [1, 26]. Generally, the biologics are well tolerated. The most common adverse effect of TNF-*α* antagonist is bacterial and fungal infection, for example, tuberculosis is common in patients receiving infliximab. Malignancy may also be associated with use of anti-TNF-*α* therapy, especially non-Hodgkin's lymphoma is reported [2].

## 7. Natural Agents 

Natural agents including flavonoids, terpenes, quinones, catechins, alkaloids, anthocyanins, and anthoxanthins are known to exhibit anti-inflammatory activity. Curcumin, resveratrol, guggulsterone, withanolide, boswellic acid, and 6-shogaol are some of the polyphenols that have been tested for the treatment of arthritis [27]. All these herbal drugs suppress the activation of nuclear factor-kB and thus lead to downregulation of the expression of TNF-*α* [28], adhesion molecules [29], metalloproteinase [30], cyclooxygenase-2 [30], 5-lipoxygenase [31], and other inflammatory intermediates [32], all of which are associated with arthritis. Curcumin has also been shown to suppress the expression of TNF-*α*-induced metalloproteinase-13 in primary chondrocytes [33]. The antiarthritic activity of curcumin has been supported by *in vitro* and *in vivo* studies [34, 35]. Withanolides, found in *Withania somnifera*, are known to be potent inhibitors of angiogenesis, inflammation, and oxidative stress [36].

The potential therapeutic agents for the treatment of RA and their sites of action are shown in Figure 1 and tabulated in Table 1.

## 8. Drug Delivery Systems for RA Therapy

A delivery system that delivers the drug directly to the synovial cavity is found to be more effective than those that are delivered systemically [37]. However, most of the current therapies for RA do not exhibit joint specificity. Therefore, to achieve effective drug concentrations in affected joints, high systemic doses of drug need to be administered, which may lead to significant systemic side effects. Reduction in drug doses may attenuate toxicity but on the other hand may lead to decreased therapeutic efficacy. To strike a balance between efficacy and side effects, several approaches have been reported that specifically target drugs to affected joints. In view of this, the novel drug delivery systems like controlled release pellets [38–40], liposomes [41], sustained release pellets [42], microspheres [43], microcapsules [44], soft gels [45], nanocomposites [46, 47], topical formulations [48], microemulsions [49], nanosuspensions [50], suppositories [51], microsponges [52], and solid dispersions [53] have been formulated. Various drugs and their delivery approaches for the effective treatment of RA are listed in Table 2.

### 8.1. Liposomes

Though many novel drug delivery systems have emerged in the last two decades for the targeted delivery of anti-rheumatoid drugs to the synovial fluid, liposomes provide an effective and convenient drug delivery capable of reducing the side effects due to following advantages [130–134].

1. Liposomes are biocompatible, completely biodegradable, nontoxic, flexible, and nonimmunogenic.
2. They offer both a lipophilic and an aqueous environment “milieu interne” in one system and are, therefore, suitable for delivery of drugs with varying solubility profiles including hydrophobic, amphipathic, and hydrophilic molecules.
3. They have the ability to protect the encapsulated drug from the external environment (Amphotericin B, Taxol).
4. They act as sustained release depots (e.g., Propranolol, Cyclosporin).
5. They can be formulated into a number of dosage forms, for example, a suspension, an aerosol, or in a semisolid form such as gel, cream, and lotion, as a dry vesicular powder (proliposome) for reconstitution.
6. They can be administered through ocular, pulmonary, nasal, oral, intramuscular, subcutaneous, topical, and intravenous routes.
7. Apart from entrapment of small molecules, liposomes are also capable of encapsulating macromolecules like superoxide dismutase, haemoglobin, erythropoietin, interleukin-2 and interferon gamma
8. They offer reduced toxicity as the exposure of nontargeted sites to the drug is reduced.
9. They alter the pharmacokinetic and pharmacodynamic profiles of drugs (e.g., reduced elimination, increased circulation life time)
10. They exhibit flexibility to couple with site-specific ligands to achieve active targeting (e.g., anticancer and antimicrobial drugs).

### 8.2. Significance of Use of Liposomes in the Delivery of Anti-Rheumatoids

**Figure 2:**
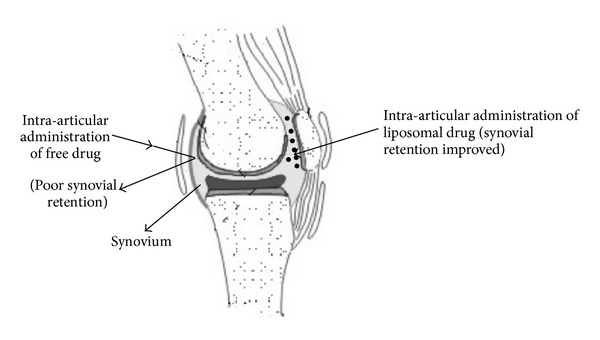
Rationale for the use of liposomes in rheumatoid arthritis.

Till date, oral administration of anti-rheumatoids for treatment of arthritis has been a consistent challenge for the clinicians, as there are severe clinical complications attached to their long-term oral use. The long-term administration of NSAIDs for the treatment of RA is associated with gastrodestructive effects that may be manifested as ulcers and intra-abdominal bleeding. Oral or intramuscular administration of steroidal drugs is generally associated with irreversible suppression of the immune system. DMARDs given by oral or intravenous or intramuscular route are known to be toxic to the immune system [130]. In order to overcome the systemic effects of these drugs, they can be directly targeted to the synovial capsule of the affected joint through intravenous route, especially when the disease manifests only in limited number of joints [135]. However, the rapid clearance of drugs from the synovial cavity into the blood stream defeats the purpose of their intra-articular administration. In this regard, liposomes have proven to be the most suitable delivery systems for retaining the drug in the synovial cavity by virtue of their size and chemical composition [130]. The clearance of intrasynovially administered drugs can be overcome through liposomes by virtue of the size of multilamellar vesicles (MLVs) [136]. This facilitates the uptake of drug by the target synovial cells and reduces the exposure to nontarget sites, eliminating the undesirable side effects. The rationale for the use of liposomes in rheumatoid arthritis is shown in Figure 2.

A number of antirheumatic drugs have been tried for the treatment of RA using liposomes as drug carrier as shown in Table 3. These are discussed below.

### 8.3. NSAIDs

A series of liposomal formulations of indomethacin have been prepared using various phospholipids. When the effect of method of preparation, lipid composition, and charge on drug retention was studied, MLVs were found to exhibit the highest drug release. Positively charged stearylamine-containing liposomes were found to slow the release of drug. This effect of charge has been attributed to electrostatic interaction (hydrogen bonding) between the acid moiety of drug and the amine moiety of lipid. The anti-inflammatory activity of indomethacin liposomes was found to be significantly higher than that of free drug in both carrageenan-induced rat paw edema and adjuvant arthritis models [79].

Various vesicular systems like liposome, niosome, lipogelosome, and niogelosome formulations were used for encapsulation of diclofenac sodium and then evaluated for drug release properties as well as *in vitro* characterization studies. Radiolabelled Tc-99m and gamma scintigraphic methods were used to evaluate the retention time of different drug delivery system for intra-articular administration. Longest retention time was observed with the radiolabelled lipogelosome formulation of diclofenac sodium [137].

In another study, diclofenac sodium-loaded lipogelosome formulation was reported to exhibit better anti-inflammatory effect after single-dose intra-articular administration as compared to topically used commercial product. Histopathological examination of synovium revealed significant lower scores for inflammatory changes after intra-articular injection of this formulation [66].

### 8.4. Glucocorticoids

A number of studies has been reported where liposomal entrapment of glucocorticoids has been shown to lead to a remarkable enhancement in the antiarthritic effect of drugs. The improvement in antiarthritic activity of glucocorticoids on entrapment in liposomes was reported for cortisol for first time [89].

The anti-inflammatory activity of cortisol palmitate liposomes was determined in rabbit knee by measuring joint temperature and diameter. Bilateral arthritis was induced by intra-articular injection of a preformed insoluble complex of poly-D-lysine and hyaluronic acid in both knee joints. The data obtained from the study revealed that the anti-inflammatory activity of liposomal cortisol palmitate was dose dependent for both the parameters of inflammation [89, 138].

Davidenkova et al., 1984, reported that hydrocortisone acetate incorporated in liposomes was found to have comparable effect with commercial drug in the form of suspension at 1/10th dose level. The study revealed that the encapsulation of drug into liposomes also prolonged the duration of action of drug [90].

Single intravenous injection (10 mg/kg) of prednisolone phosphate encapsulated in long-circulating PEG-liposomes was more effective in reducing both joint inflammation and cartilage destruction as compared to free drug in mice with collagen type-II and adjuvant-induced arthritis. The free drug at the same dose was reported to be much less effective even after repeated daily injections [91].

Harigai et al., 2007, reported that the prednisolone phosphate liposomes containing 3,5-dipentadecyclobenzaamidine hydrochloride (TRX-20) inhibited the production of inflammatory cytokines (IL-6) and chemokines (IL-8) more effectively than prednisolone phosphate-containing liposomes without TRX-20. The TRX-20 also increased the affinity of liposomes towards human fibroblast-like synovial cells. This combined delivery of drugs through liposomes was proposed as an approach to enhance the clinical use of glucocorticoids for treating RA [139].

Sterically stabilized (pegylated) nanoliposomes of amphipathic weak acid prodrugs of glucocorticoids (methyl prednisolone hemisuccinate and betamethasone hemisuccinate) were prepared and evaluated for their antiarthritic potential in Lewis rats and Beagle dogs by Avnir et al., 2008. The authors reported that the liposomal formulation exhibited high encapsulation efficacy (94%) and a high drug-lipid mole ration (0.41). The therapeutic efficacy of liposomal formulation was also reported to be superior to that of free glucocorticoids in arthritic rats, both at an early disease stage and at the peak of the disease [92].

Liposomal prednisolone phosphate strongly suppressed knee joint swelling, synovial infiltration, and bone erosion in antigen-induced arthritis. The suppression of bone erosion is likely to be mediated by inhibition of osteoclast activity via suppression of osteoclast differentiation factors and/or by directly blocking differentiation of macrophage-like precursor cells into functional osteoclasts [93].

In another study by the same authors, the effect of single injection of liposomal formulation of prednisolone phosphate on metalloproteases and aggrecanases mediated cartilage destruction in antigen-induced arthritis was studied in comparison to free prednisolone phosphate. The synovial immune cell infiltration was found to be less in mice treated with prednisolone phosphate-liposomes as compared to control group. Liposomal formulation also significantly suppressed interleukin 1*β*, proteases, metalloproteases-3, and aggrecanases in the synovium, thereby suppressing the destruction of cartilage matrix in antigen-induced arthritis [94].

The anti-inflammatory effect of sterically stabilised nanoliposomes of methyl prednisolone hemisuccinate and betamethasone hemisuccinate was analysed in adjuvant arthritis by Ulmansky et al. Both nano-liposome formulations suppressed arthritis significantly, compared to higher doses of free drug or TNF-*α* antagonists (infliximab, etanercept). Glucocorticoid nanoliposomes also suppressed the secretion of proinflammatory cytokines without any effect on TGF-*α* level [95].

Liposome entrapped dexamethasone palmitate was compared for its pharmacokinetic and therapeutic effect to microcrystalline triamcinolone acetonide by Bonanomi et al., 1987. Joint circumference was observed to be decreased significantly in rabbits administered with dexamethasone palmitate as compared to triamcinolone acetonide. It was also observed that about 36% of the liposomal dexamethasone palmitate was still in the synovial fluid after 6 h of injection while triamcinolone acetonide had fully disappeared from the joints till that time. Increase in diameter of liposomal vesicles was shown to improve the retention time of drug [96].

Intra-articular injection of multilamellar and oligolamellar liposomal vesicles containing dexamethasone palmitate were investigated for bioavailability studies. The bioavailability of drug from oligolamellar vesicles was found to be more as compared to that from multilamellar vesicles [97].

Dexamethasone phosphate containing arginine-glycine-aspartic acid peptide polyethylene glycol liposomes was screened for specific binding to *αvβ*3 integrins expressed on angiogenic vascular endothelial cells at the site of inflammation. The formulated liposomes targeted vascular endothelial cells at the site of inflammation and resulted in strong, long-lasting antiarthritic effect in rat with antigen-induced arthritis [98].

Glucocorticoid dexamethasone phosphate encapsulated in large non-PEGylated liposomes exhibited potent anti-inflammatory activity as compared to free drug in rat antigen-induced arthritis. It was observed that the intravenous injection (i.v.) of non-PEGylated liposomal drug completely suppressed joint swelling [99].

In 2009, Rauchhaus et al. compared the therapeutic efficacy of liposomal dexamethasone phosphate with free dexamethasone in mouse collagen-induced arthritis. Single intravenous injection of 4 mg/kg liposomal formulation produced a significant therapeutic effect for at least 7 days. On the other hand, single administration with free dexamethasone was not found to be very effective and multiple injections were required [100].

The efficacy of i.v. injection of liposomally encapsulated dexamethasone phosphate was evaluated in comparison to that of free drug in rats with established adjuvant arthritis. Liposomal-dexamethasone phosphate suppressed haematological signs of arthritis including erythrocyte sedimentation rate, white blood cell count, circulating antimycobacterial IgG, and production of IL-1 and IL-6 by macrophages in a dose-dependent manner for dosage between 0.01 and 1.0 mg/kg. The effects of medium dose of liposomal formulation were found to be equal (in short term) or superior (in long term) to those of high dose of free drug. The residence time of liposomal drug was significantly higher in synovial membrane than that of the free drug even after 48 hours of last injection [101].

The therapeutic activity and adverse effects of three different glucocorticoids (dexamethasone, budesonide, and prednisolone) encapsulated in long circulating liposomes was determined in rats with adjuvant arthritis and collagen-induced arthritis. Encapsulation of drugs in liposomes not only increased their therapeutic efficacy but also decreased their clearance from the body [102].

Intra-articular injection of triamcinolone acetonide-21-palmitate incorporated liposomes was studied for its efficacy in arthritis using rabbits by Lopez-Garcia et al. 1993., The liposomal formulation was more effective as compared to free triamcinolone acetonide in suppressing arthritis. Moreover, the retention time was also found to be greater for liposomal formulation [103].

### 8.5. DMARDs

Small unilamellar vesicles (SUVs) of sodium aurothiomalate were prepared and evaluated for anti-inflammatory action in collagen-induced arthritis as compared to free drug. Intramuscular injection of SUVs was found to cause 50% reduction in symptoms. SUVs of sodium aurothiomalate also inhibited cellular infiltration of lymphocytes into synovia of collagen treated mice as confirmed by histological examination [11].

Retention and distribution of liposome-entrapped methotrexate were evaluated in antigen-induced arthritic rabbit joints in comparison to those of free methotrexate. About 79% of free methotrexate was rapidly cleared from joint within 24 hours of intra-articular injection, while at the same time about half of the liposomal-entrapped drug (45%) was recovered from the joint. Although the uptake of liposomes by inflamed synovium was lower than expected, it was found to be 40 times higher than that with free methotrexate [104].

Methotrexate liposomes suppressed the joint swelling and rise in temperature in antigen-induced arthritic rabbits. Liposomal formulation was even effective after 7 days of antigen challenge at one-tenth dose as compared to free methotrexate. Decrease in synovial hyperplasia, cellular infiltration, and cartilage erosion was observed with liposomal methotrexate [105].

The efficacy of free and liposomally conjugated methotrexate was compared in rats with adjuvant-induced arthritis. Methotrexate suppressed but did not abolish the development of joint inflammation when the treatment was started on the day of arthritis induction. Methotrexate liposome, thus, has significant anti-inflammatory effect on established arthritis [106].

Multilamellar vesicles of methotrexate exhibited a significant anti-inflammatory effect compared to free methotrexate and methotrexate entrapped in small unilamellar vesicles in Lewis rats with antigen-induced arthritis, after single intra-articular injection. The multilamellar vesicles were found to inhibit the cellular infiltration associated with arthritis [107].

In another study, liposomes of methotrexate with conventional and long-circulation times were prepared and their therapeutic efficacy was assessed using the rat collagen-induced arthritis. Both types of liposomes inhibited the release of IL-1*β* from macrophages in a dose-dependent manner while free methotrexate had no effect on release of mediators. In short-term treatment, conventional liposomes showed greater anti-inflammatory activity than long-circulation liposomes. However, in long-term, liposomal preparation with extended circulation time also exerted potent anti-inflammatory effects in rat arthritis [108].

Intravenous injections of methotrexate liposomes were proven to be powerful inhibitors of both IL-1*β* and PGE_2_ release form macrophages in collagen-induced arthritis. Polyethyleneglycol-liposomes with long-circulation times did not appear to have much therapeutic potential for treating arthritis *in vivo* [109].

Williams et al., 2001, reported that single intra-articular injection of liposomally conjugated methotrexate significantly reduced knee swelling (1.94 ± 0.12 mm) as compared to free drug (3.17 ± 0.18 mm) in antigen-induced arthritis in rats. This anti-inflammatory effect was accompanied by inhibition of both IL-1*β* and IL-6 mRNA expression in synovial tissue. Liposomal treatment also inhibited the progression of antigen-induced arthritis [110].

The efficacy of chitosan-coated conventional liposomes and PEGylated liposomes of methotrexate was compared to that of the uncoated conventional liposomes in Wistar-Lewis rats with Freund's adjuvant arthritis. Chitosan coating was found to increase both the physical stability and entrapment efficiency. Both chitosan-coated and PEGylated liposomes exhibited significant anti-inflammatory activity and released the drug for longer period of time than uncoated conventional liposomes [61].

The toxicity of methotrexate loaded liposomes was compared with methotrexate injectable solution in rat adjuvant arthritis. Results of the haematological and biochemical tests revealed that methotrexate loaded liposomes showed reduced toxicity as compared to injectable methotrexate [111].

Depletion of phagocytic synovial lining cells by single intra-articular injection of clodronate encapsulated liposomes was found to significantly reduce the joint swelling, as compared to normal nondepleted joints in rats with antigen-induced arthritis [112].

In another study, clodronate-laden liposomes were reported to suppress the clinical signs of inflammation for longer period of time in rats with adjuvant arthritis and antigen-induced arthritis than the uncapsulated drug. A significant reduction in macrophages was observed not only in synovial membrane, but also in liver and spleen [114, 115].

Van Lent et al. investigated the effect of local removal of phagocytic synovial lining cells from the knee joint on development of cartilage destruction in collagen type II arthritic model. In synovial lining cells depleted arthritic joint, chondrocyte death was significantly decreased. Although local clodronate liposome treatment had some beneficial effects on cartilage destruction, it was found to be more effective in presence of dexamethasone [113].

However, similar results could not be substantiated in sheep model of antigen-induced arthritis. The effect of intravenous administration of clodronate liposomes was investigated in sheep with antigen-induced arthritis. In both treatment and control group, no difference in joint diameter was observed. Moreover, both groups showed joint swelling which persisted until the end of the study [120].

A comparative study of small unilamellar and large multilamellar vesicles of clodronate was conducted in rats with antigen-induced arthritis. SUVs were found to be more effective than MLVs in reducing inflammation and joint destruction due to significant depletion of macrophages from synovial membrane [116]. In another study, single intra-articular injection of clodronate unilamellar liposomes significantly decreased synovial lining macrophages in patients with longstanding RA. Liposomal administration also decreased the expression of adhesion molecules in the cell lining. Depletion of macrophages ultimately reduced the cartilage destruction in chronic arthritis [117].

The effect of repeated intra-articular administration of low doses (0.145 mg/injection) of liposomal clodronate was investigated on established antigen-induced arthritis in rabbits. Liposomal clodronate treated rabbits showed reduction in joint swelling even after first three injections. Moreover, the levels of macrophages were found to be low in the synovium of treated rabbits. Liposomes were detected within the joints for a period as long as one week after injection which explained the sustained action of drug for longer period of time [118].

Richards et al. reported that single intravenous injection of 20 mg of clodronate encapsulated in SUVs significantly suppressed the development of chronic streptococcal cell wall-induced arthritis in Lewis rats. Administration of liposomal formulation was found to significantly deplete the macrophages which, in turn, inhibited the production of proinflammatory cytokines and ultimately the progression of disease [119].

### 8.6. Miscellaneous Therapeutic Agents

#### 8.6.1. Superoxide Dismutase (SOD)

Intramuscular injection of liposomal bovine copper superoxide dismutase in humans was found to significantly ameliorate the clinical signs of rheumatoid arthritis [126].

In another study, the effect of size of liposomes for targeting SOD to arthritic sites was investigated after subcutaneous administration. It was observed that the uptake of small size liposomes (mean size 110 nm) was 17 times higher than that of large sized liposomes (mean size 450 nm) in the inflamed foot of rats. Small size SOD liposomes showed significantly higher anti-inflammatory activity than large sized liposomes after subcutaneous (s.c) administration which was found to be as effective as i.v. injection. Large sized liposomes were found to be more active by i.v. route as compared to s.c. route [124].

Superoxide dismutase entrapped long-circulating liposomes were prepared by different preparation protocols such as film hydration, freeze-thawing and dehydration-rehydration methods. The prepared liposomes were characterised in terms of entrapment efficiency, size, enzymatic activity, and protein structure. Two different SOD-liposomes that is, stearylamine (SA)-liposomes and polyethylene glycol (PEG)-liposomes were selected for *in vivo* evaluation using rat adjuvant arthritis model. Both PEG-liposomes and stearylamine-liposomes showed superior therapeutic activity as compared to free SOD, while PEG-liposomes exhibited stronger anti-inflammatory effects than SA-liposomes in both single dose and multiple dose-response studies [121, 122].

The comparative anti-inflammatory effect of liposomal and a tranferosomal formulation of superoxide dismutase (SOD) was determined in adjuvant-induced arthritis in rats. The amelioration of disease symptoms on animals treated with transfersomes showed that epicutaneous application of SOD had a significant role in reduction of inflammation. Secondly, transfersomes have an additional advantage that they are administered by noninvasive route [123].

The biological behaviour of acylated superoxide dismutase inserted in lipid bilayer of liposomes was investigated in comparison with SOD located in aqueous environment of liposomes in rat model of adjuvant arthritis. Acylated superoxide dismutase exhibited faster anti-inflammatory effect than SOD liposomes [125, 140].

### 8.7. Lactoferrin

Residence time of human lactoferrin entrapped in positively or negatively charged liposomes was reported in mice joints with collagen-induced arthritis. After 2 hours of intra-articular injection, 60% of the injected dose was found to be retained in the joints in case of positive liposomes and only 16% for negative pH-sensitive liposomes [127].

Trif et al. reported that multivesicular liposomes of anti-inflammatory glycoprotein, lactoferrin exhibited pronounced anti-inflammatory effect as compared to free protein in collagen-induced arthritis. Single intra-articular injection of liposomal formulation significantly decreased arthritic score for two weeks while free lactoferrin was effective only for 3-4 days. Liposomal lactoferrin also reduced proinflammatory (TNF) and increased anti-inflammatory (IL-10) cytokine production [128]

### 8.8. Boron Neutron Capture Therapy

Liposomal drug delivery system has been explored for selective delivery of boron-10 isotope to the synovial tissue in rats with collagen-induced arthritis. Intravenous injection of liposome suspension was given to Louvain rats with collagen-induced arthritis and tissue concentration of boron was determined by atomic emission spectroscopy. The final concentration of boron in synovium was found to be 22 *μ*g per gram of tissue and the highest synovium/blood boron ratio was 3 [129].

## 9. Conclusion

Arthritis, an inflammation of the joints, is a chronic disease that results from dysregulation of proinflammatory cytokines (e.g., tumor necrosis factor and interleukin-1*β*) and proinflammatory enzymes that mediate the production of prostaglandins (e.g., cyclooxygenase-2) and leukotrienes (e.g., lipoxygenase), together with the expression of adhesion molecules and matrix metalloproteinase, and hyperproliferation of synovial fibroblasts. The current treatments of RA include four categories: nonsteroidal anti-inflammatory drugs (NSAIDs), glucocorticoids, nonbiologic disease-modifying antirheumatic drugs (DMARDs), and biologic DMARDs. Moreover, numerous agents derived from plants can suppress these cell signalling intermediates, including curcumin, resveratrol, tea polyphenols, genistein, quercetin, silymarin, guggulsterone, boswellic acid, and withanolides. Though several efforts have been made, a cure for rheumatoid arthritis is yet to be discovered. As mentioned earlier, most of the current therapies for RA do not have joint specificity. Therefore, to reach effective drug concentrations in affected joint tissues, high systemic doses of drug must often be administered, which may lead to significant adverse systemic side effects; reduction in drug doses may attenuate toxicity but may lead to decreased therapeutic efficacy. To overcome this limitation, approaches that specifically target agents to affected joints offer unique promise. Liposomes have the capacity to be used as delivery and targeting agents for the administration of drugs at lower doses with reduced toxicity. With improvements in liposomal formulation antirheumatic and targeted synovial delivery, liposomes offer increased therapeutic activity and improvement in the risk-benefit ratio. Several liposomal formulations of NSAIDs, Glucocorticoids, and DMARDs have been prepared; however, their safety, stability, and efficacy are still questionable. In order to launch them effectively into market, liposomes have to pass through several clinical trials. Recent research into synovial targets and improved liposomal formulations continues to improve the use of liposomes for targeted delivery. The journey of liposomal anti-cancer drug delivery, though about 20-year long, resulted in successful culmination as a number of formulations of daunorubicin and doxorubicin are available in the market for clinical use [141]. Similar is the case with antifungal agent amphotericin B [142]. We hope for a similar kind of successful culmination of all the cited works carried out on liposomal delivery of antiarthritic drugs.

## Figures and Tables

**Table 1:** Molecular targets of antirheumatic therapeutic agents and their complications.

S. No.	Therapeutic agents	Molecular targets	Complications with long-term therapy	References
1	NSAIDs (First line therapy), for example, Ibuprofen, Naproxen, Indomethacin, Ketoprofen, Diclofenac sodium, Meloxicam	COX-2 (Non selective)	(1) Peptic ulcers (2) Dyspepsia (3) Anorexia (4) Abdominal pain (5) Nausea (6) Flatulence (7) Diarrhoea (8) Renal ulcers (9) Myocardial infarction	[6, 18, 19, 21]
2	Injectable corticosteroids	COX-2	(1) Skin atrophy	[5, 6]
3	DMARDs, for example, Gold salts (Aurothiomalate), Leflunomide, Sulfasalazine, Methotrexate, Azathioprine, Minocycline, Hydroxychloroquine, Cyclosporine	TNF-*α*, IL	(1) Digestive organ dysfunction (2) Liver dysfunction (3) Kidney dysfunction (4) Stomatitis (5) Depilation and myelosuppression	[22, 25]
4	Coxibs, for example, Celecoxib, Etoricoxib	COX-2 (Selective coxib)	(1) Peptic ulcers	[6]
5	Glucocorticoids, for example, Prednisone, Methyl prednisone, Hydrocortisone, Dexamethasone, Betamethasone	COX-2	(1) Impaired wound healing (2) Skin atrophy (3) Osteoporosis (4) Muscle atrophy (5) Cataract (6) Glaucoma (7) Peptic ulcer (8) Manifestation of latent diabetes (9) Premature mortality	[5, 6]
6	Biologics	TNK-*α*, IL-1, IL-6	(1) Malignancy (2) Tuberculosis	[2]
7	Natural products, for example Curcumin, Resveratrol, Guggulsterone, Withanolide, and so forth.	NF-*κ*B, COX-2,5-LOX, TNF-*α*, IL-1*β*, IL-6, IL-8, MMPs	Not reported	[27]

**Table 2:** Various drugs and their delivery approaches for the effective treatment of rheumatoid arthritis.

Drug	Delivery systems	Key observation	References
Corticosteroids			
Prednisolone	Liposomes	Tissue targeting	[54]
	Microspheres	Prolonged release	[55]
	Nanoparticles	Improved efficacy	[56]

DMARDS (Disease modifying antirheumatic drugs)			
Sod. aurothiomalate	Liposomes	Better safety profile and prolonged action	[11]
Azathioprine	Sustain release tablets	Better patient safety	[57]
Leflunomide	Microspheres	Rapid action	[58]
	Microcapsules	Sustained action	[59]
Methotrexate	Multilamellar vesicles	Increased permeation	[60]
	Liposomes	Drug targeting, prolonged therapeutic effect	[61]
	Microspheres	Retention of drug in joints and less clearance into blood	[62]
	Encapsulated lipid based drug-delivery	Prolonged half-life, extended drug release	[63]
Tacrolimus	Liposomes	Improved oral delivery	[64]

NSAIDS (Nonsteroidal anti-inflammatory drugs)			
Diclofenac	Sustained release pellets	Less side effects	[65]
	Lipogelosomes	Less side effects, Improved efficacy	[66]
	Pharmacosomes	Improved solubility Lower gastrointestinal toxicity	[67]
	Microcapsules	Sustained release	[68]
	Microspheres	Long therapeutic effect	[69]
	Nanoparticles	Prolonged drug release	[47]
	Suppositories	Improved efficacy	[70]
Ibuprofen	Microemulsions	Increased skin permeation, Increased oral bioavailability	[71, 72]
	Microspheres	Prolonged therapeutic effect	[73]
	Transfersome	Prolonged therapeutic effect and good stability	[74]
	Sustained release formulation	Prolonged therapeutic effect and improved patient compliance	[75]
Indomethacin	Slow released formulations	Better safety and controlled release characteristics	[76, 77]
	Dendrimers	Targeted delivery	[78]
	Liposomes	More effective and minimum side effects	[79, 80]
	Microballoons	Good floating ability	[81]
	Microspheres	Improved targeting	[82]
	Nanoemulsions	Improved bioavailability through transdermal delivery	[83]
	Suppositories	Enhanced therapeutic efficacy	[84]
Ketoprofen	Transdermal patch	Improved skin permeation	[85]
	Microspheres	Prolonged therapeutic effect	[86]
	Microcapsules	Optimum sustained release	[87]
	Nanoemulsions	Enhanced skin permeation	[88]

**Table 3:** Liposomal drug formulations in treatment of Rheumatoid arthritis.

S. No	Drug	Liposomal type	Animal used	Animal model	Route of administration	Observed effect	Reference
1	Indomethacin	Large unilamellar vesicles	Rat	Carrageenan induced paw edema and Adjuvant arthritis	Intra-peritoneal	Increase anti-inflammatory activity, less ulcer index	[79]
2	Diclofenac sodium	Lipogelosome	Rabbit	Antigen-induced arthritis	Intra-articular	Reduce side effects, increase retention of drug at inflammatory site	[6, 66]
3	Cortisol palmitate	Not defined	Rabbit	Poly-D-lysine and hyaluronic acid complex injection	Intra-articular	Reduce temperature and diameter in arthritic joints	[89]
4	Hydrocortisone	Multilamellar liposomes	Rabbit	Antigen-induced arthritis	Intra-articular	Prolong anti-inflammatory effect	[90]
5	Prednisolone phosphate	PEG-liposomes	Mice	Collagen type-II and adjuvant-induced arthritis	Intravenous	Reduce cartilage damage	[91]
6	Methyl prednisolone hemisuccinate	Nanoliposomes	Lewis rat, Beagle dog	Adjuvant arthritis	Intravenous	High encapsulation efficacy, high drug-lipid mole ration, increase therapeutic efficacy	[92]
**7**	Prednisolone phosphate	Not defined	Mice	Antigen-induced arthritis	Intravenous	Suppression of bone erosion, less synovial immune cell infiltration, Suppress metalloproteases and aggrecanases in synovium	[93, 94]
8	Methyl prednisolone hemisuccinate	Nanoliposomes	Lewis rat	Adjuvant arthritis	Intravenous or subcutaneous	Reduce arthritis, suppression of secretion of proinflammatory cytokines	[95]
9	Betamethasone hemisuccinate	Nanoliposomes	Lewis rat	Adjuvant arthritis	Intravenous or subcutaneous	Reduce arthritis, suppression of secretion of proinflammatory cytokines	[95]
10	Dexamethasone phosphate	Oligolamellar and multilamellar vesicles	Rabbit	Antigen-induced arthritis	Intra-articular	Increase retention of drug in synovium and synovial fluid	[96, 97]
11	Dexamethasone phosphate	RGD-PEG-Liposomes	Lewis rat	Antigen-induced arthritis	Intravenous	Strong and long-lasting antiarthritic effect, specifically target vesicular endothelial sites at site of inflammation	[98]
12	Dexamethasone phosphate	Non-PEGlyated liposomes	Rat	Antigen-induced arthritis	Intravenous	Suppress joint swelling	[99]
13	Dexamethasone phosphate	Non-PEGlyated liposomes	Mouse	Collagen induces arthritis	Intravenous	Persistent anti-inflammatory effect, suppression of hypothalamic-pituitary	[100]
14	Dexamethasone phosphate	Not defined	Lewis rat	Adjuvant arthritis	Intravenous	Suppression of histological signs of arthritis, increased residence time of drug in synovial membrane	[101]
15	Dexamethasone, budesonide, prednisolone	Long circulating liposomes	Rat	Adjuvant arthritis, collagen-induced arthritis	Intravenous	Increase therapeutic efficacy, decrease clearance of drug from body	[98, 102]
16	Triamcinolone	Not defined	Rabbit	Carrageenan-induced paw edema	Intra-articular	Effectively suppress arthritis, longer retention of drug in articular cavity	[103]
17	Sodium aurothiomalate	Small unilamellar vesicles	Mice	Collagen induces arthritis	Intra-muscular	Inhibit cellular infiltration of lymphocytes into the synovium, reduction in arthritis symptoms	[11]
18	Methotrexate	Not defined	Rabbit	Antigen-induced arthritis	Intra-articular	Long retention of drug in joints, suppressed joint swelling and rise in temperature, Decrease in synovial hyperplasia, cellular infiltration and cartilage erosion	[104, 105]
19	Methotrexate	Small unilamellar vesicles	Rat	Adjuvant-induced arthritis	Intravenous	Significant anti-inflammatory effect	[106]
20	Methotrexate	Multilamellar vesicles	Rat	Antigen-induced arthritis	Intra-articular	Significant anti-inflammatory effect, Inhibit cellular infiltration	[107]
21	Methotrexate	Small unilamellar vesicles	Rat	Collagen induces arthritis	Intravenous	Inhibit the release of IL-1*β* from macrophages, potent anti-inflammatory activity	[108]
22	Methotrexate	PEG-liposomes	Rat	Collagen induces arthritis	Intravenous	Inhibitors release of both IL-1*β* and PGE2 form macrophages	[109]
23	Methotrexate	Large multilamellar vesicles	Rat	Antigen-induced arthritis	Intra-articular	Inhibition of both IL-1*β* and IL-6 mRNA expression in synovial tissue, reduce knee swelling, Inhibit progression of antigen-induced arthritis	[110]
24	Methotrexate	PEGylted liposomes	Wistar-Lewis rat	Adjuvant arthritis	Intravenous	Increased physical stability and entrapment efficacy, significant anti-inflammatory activity	[61]
25	Methotrexate	Not defined	Wistar Rat	Adjuvant arthritis	Intravenous	Reduced toxicity	[111]
26	Clodronate	Not defined	Mice	Collagen induces arthritis	Intra-articular	Reduced joint swelling, significantly decreased chondrocyte death, Reduced cartilage destruction	[112, 113]
27	Clodronate	Multilamellar vesicles	Rat	Adjuant arthritis, antigen-induced arthritis	Intravenous	Reduction of macrophages in synovial membrane, liver, and spleen, reduced inflammation and joint destruction	[114–116]
28	Clodronate	Unilamellar liposomes	Human	RA patients	Intra-articular	Decreased synovial lining macrophages and expression of adhesion molecules, reduced cartilage destruction	[117]
29	Clodronate	Not defined	Rabbit	Antigen-induced arthritis	Intra-articular	Low level of macrophages in synovium, reduction in joint swelling, sustained action of drug	[118]
30	Clodronate	Small unilamellar vesicles	Lewis rat	Streptococcal cell wall—induced arthritis	Intravenous	Depletion of macrophages, inhibited the production of proinflammatory cytokines, decreased progression of disease	[119]
31	Clodronate	Multilamellar vesicles	Sheep	Antigen-induced arthritis	Intravenous	No significant anti-inflammatory effect	[120]
32	Superoxide dismutase	Stearylamine and PEG liposomes	Wistar rat	Antigen-induced arthritis	Intravenous	Potent anti-inflammatory activity	[121, 122]
33	Superoxide dismutase	Liposomes and transfersomes	Wistar rat	Adjuvant arthritis	Epicutaneous	Significant reduction in inflammation	[123]
34	Superoxide dismutase	Not defined	Rat	Adjuvant arthritis	Subcutaneous	Significant anti-inflammatory activity	[124]
35	Superoxide dismutase	Multilamellar and PEGylated liposomes	Wistar rat	Adjuvant arthritis	Intravenous	Faster anti-inflammatory activity	[125]
36	Superoxide dismutase	Not defined	Human	Human RA	Intramuscular	Significant improvement in clinical signs of inflammation	[126]
37	Lactoferrin	Not defined	Mice	Collagen-induced arthritis	Intra-articular	Increased retention of drug in joints, reduced proinflammatory (TNF) and increased anti-inflammatory (IL-10) cytokine production	[127, 128]
38	Boron neutron capture therapy	Not defined	Louvain rat	Collagen-induced arthritis	Intravenous	High concentration of boron in synovium	[129]

## References

[1] Ban A, Inaba M, Furumitsu Y (2010). Time-course of health status in patients with rheumatoid arthritis during the first year of treatment with infliximab. *Biomedicine and Pharmacotherapy*.

[2] Yen J-H (2006). Treatment of early rheumatoid arthritis in developing countries. Biologics or disease-modifying anti-rheumatic drugs?. *Biomedicine and Pharmacotherapy*.

[3] Brasington RD, Kahl LE, Ranganathan P, Latinis KM, Velazquez C, Atkinson JP (2003). Immunologic rheumatic disorders. *Journal of Allergy and Clinical Immunology*.

[4] Furst DE (2004). Anakinra: review of recombinant human interleukin-I receptor antagonist in the treatment of rheumatoid arthritis. *Clinical Therapeutics*.

[5] Russell A, Haraoui B, Keystone E, Klinkhoff A (2001). Current and emerging therapies for rheumatoid arthritis, with a focus on infliximab: clinical impact on joint damage and cost of care in Canada. *Clinical Therapeutics*.

[6] Yuan F, Quan L-D, Cui L, Goldring SR, Wang D (2012). Development of macromolecular prodrug for rheumatoid arthritis. *Advanced Drug Delivery Reviews*.

[7] Bonafede MMK, Fox KM, Johnson BH, Watson C, Gandra SR (2012). Factors associated with the initiation of disease-modifying antirheumatic drugs in newly diagnosed rheumatoid arthritis: a retrospective claims database study. *Clinical Therapeutics*.

[8] Marks WH (2011). *Tripterygium wilfordii* Hook F. versus Sulfasalazine in the treatment of rheumatoid arthritis: a well-designed clinical trial of a botanical demonstrating effectiveness. *Fitoterapia*.

[9] Crielaard BJ, Lammers T, Schiffelers RM, Storm G (2012). Drug targeting systems for inflammatory disease: one for all, all for one. *Journal of Controlled Release*.

[10] Zintzaras E, Dahabreh IJ, Giannouli S, Voulgarelis M, Moutsopoulos HM (2008). Infliximab and methotrexate in the treatment of rheumatoid arthritis: a systematic review and meta-analysis of dosage regimens. *Clinical Therapeutics*.

[11] Konigsberg PJ, Debrick JE, Pawlowski TJ, Staerz UD (1999). Liposome encapsulated aurothiomalate reduces collagen-induced arthritis in DBA/1J mice. *Biochimica et Biophysica Acta*.

[12] Herrmann ML, Schleyerbach R, Kirschbaum BJ (2000). Leflunomide: an immunomodulatory drug for the treatment of rheumatoid arthritis and other autoimmune diseases. *Immunopharmacology*.

[13] Massarotti EM (2008). Clinical and patient-reported outcomes in clinical trials of abatacept in the treatment of rheumatoid arthritis. *Clinical Therapeutics*.

[14] Curtis JR, Singh JA (2011). Use of biologics in rheumatoid arthritis: current and emerging paradigms of care. *Clinical Therapeutics*.

[15] Larsen C, Østergaard J, Larsen SW (2008). Intra-articular depot formulation principles: role in the management of postoperative pain and arthritic disorders. *Journal of Pharmaceutical Sciences*.

[16] van den Hoven JM, van Tomme SR, Metselaar JM, Nuijen B, Beijnen JH, Storm G (2011). Liposomal drug formulations in the treatment of rheumatoid arthritis. *Molecular Pharmaceutics*.

[17] Gulati M, Singh S, Chopra D, Duggal S, Kumar R (2008). Use of liposomal drugs in the treatment of rheumatoid arthritis. *Current Rheumatology Reviews*.

[18] Edwards JE, McQuay HJ, Moore RA (2004). Efficacy and safety of valdecoxib for treatment of osteoarthritis and rheumatoid arthritis: systematic review of randomised controlled trials. *Pain*.

[19] Scheiman JM, Hindley CE (2010). Strategies to optimize treatment with NSAIDs in patients at risk for gastrointestinal and cardiovascular adverse events. *Clinical Therapeutics*.

[20] Schnitzer TJ, Truitt K, Fleischmann R (1999). The safety profile, tolerability, and effective dose range of rofecoxib in the treatment of rheumatoid arthritis. *Clinical Therapeutics*.

[21] Krug H, Broadwell LK, Berry M, Delapp R, Palmer RH, Mahowald M (2000). Tolerability and efficacy of nabumetone and naproxen in the treatment of rheumatoid arthritis. *Clinical Therapeutics*.

[22] Jiang M, Zha Q, He Y, Lu A (2012). Risk factors of gastrointestinal and hepatic adverse drug reactions in the treatment of rheumatoid arthritis with biomedical combination therapy and Chinese medicine. *Journal of Ethnopharmacology*.

[23] Chen Z, Li XP, Li ZJ, Xu L, Li XM (2013). Reduced hepatotoxicity by total glucosides of paeony in combination treatment with leflunomide and methotrexate for patients with active rheumatoid arthritis. *International Immunopharmacology*.

[24] Schattenkirchner M (2000). The use of leflunomide in the treatment of rheumatoid arthritis: an experimental and clinical review. *Immunopharmacology*.

[25] Ichihara H, Hino M, Makizono T, Umebayashi M, Matsumoto Y, Ueoka R (2011). Inhibitory effects of hybrid liposomes on the growth of synoviocyte causing rheumatoid arthritis. *Bioorganic and Medicinal Chemistry Letters*.

[26] DeWitt EM, Lin L, Glick HA, Anstrom KJ, Schulman KA, Reed SD (2009). Pattern and predictors of the initiation of biologic agents for the treatment of rheumatoid arthritis in the United States: an analysis using a large observational data bank. *Clinical Therapeutics*.

[27] Khanna D, Sethi G, Ahn KS (2007). Natural products as a gold mine for arthritis treatment. *Current Opinion in Pharmacology*.

[28] Shishodia S, Amin HM, Lai R, Aggarwal BB (2005). Curcumin (diferuloylmethane) inhibits constitutive NF-*κ*B activation, induces G1/S arrest, suppresses proliferation, and induces apoptosis in mantle cell lymphoma. *Biochemical Pharmacology*.

[29] Kumar A, Dhawan S, Hardegen NJ, Aggarwal BB (1998). Curcumin (diferuloylmethane) inhibition of tumor necrosis factor (TNF)- mediated adhesion of monocytes to endothelial cells by suppression of cell surface expression of adhesion molecules and of nuclear factor-*κ*B activation. *Biochemical Pharmacology*.

[30] Aggarwal S, Ichikawa H, Takada Y, Sandur SK, Shishodia S, Aggarwal BB (2006). Curcumin (diferuloylmethane) down-regulates expression of cell proliferation and antiapoptotic and metastatic gene products through suppression of I*κ*B*α* kinase and Akt activation. *Molecular Pharmacology*.

[31] Skrzypczak-Jankun E, Zhou K, McCabe NP, Selman SH, Jankun J (2003). Structure of curcumin in complex with lipoxygenase and its significance in cancer. *International Journal of Molecular Medicine*.

[32] Aggarwal BB, Kumar A, Bharti AC (2003). Anticancer potential of curcumin: preclinical and clinical studies. *Anticancer Research*.

[33] Liacini A, Sylvester J, Li WQ (2003). Induction of matrix metalloproteinase-13 gene expression by TNF-*α* is mediated by MAP kinases, AP-1, and NF-*κ*B transcription factors in articular chondrocytes. *Experimental Cell Research*.

[34] Joe B, Rao UJSR, Lokesh BR (1997). Presence of an acidic glycoprotein in the serum of arthritic rats: modulation by capsaicin and curcumin. *Molecular and Cellular Biochemistry*.

[35] Funk JL, Oyarzo JN, Frye JB (2006). Turmeric extracts containing curcuminoids prevent experimental rheumatoid arthritis. *Journal of Natural Products*.

[36] Ichikawa H, Takada Y, Shishodia S, Jayaprakasam B, Nair MG, Aggarwal BB (2006). Withanolides potentiate apoptosis, inhibit invasion, and abolish osteoclastogenesis through suppression of nuclear factor-*Κ*B (NF-*Κ*B) activation and NF-*Κ*B-regulated gene expression. *Molecular Cancer Therapeutics*.

[37] Quan L-D, Thiele GM, Tian J, Wang D (2008). The development of novel therapies for rheumatoid arthritis. *Expert Opinion on Therapeutic Patents*.

[38] Vanniasinghe AS, Bender V, Manolios N (2009). The potential of liposomal drug delivery for the treatment of inflammatory arthritis. *Seminars in Arthritis and Rheumatism*.

[39] Gabard B, Ellgehausen P (1993). Comparative clinical trial with immediate-release diclofenac pellets, 50 mg TID, and slow-release diclofenac pellets, 75 mg bid. *Current Therapeutic Research*.

[40] González-Rodríguez ML, Maestrelli F, Mura P, Rabasco AM (2003). In vitro release of sodium diclofenac from a central core matrix tablet aimed for colonic drug delivery. *European Journal of Pharmaceutical Sciences*.

[41] Vyas SP, Singh R, Asati RK (1995). Liposomally encapsulated diclofenac for sonophoresis induced systemic delivery. *Journal of Microencapsulation*.

[42] Kramar A, Turk S, Vrečer F (2003). Statistical optimisation of diclofenac sustained release pellets coated with polymethacrylic films. *International Journal of Pharmaceutics*.

[43] Lewis L, Boni R, Adeyeye CM (1998). Effect of emulsifier blend on the characteristics of sustained release diclofenac microspheres. *Journal of Microencapsulation*.

[44] Biju SS, Saisivam S, Rajan NSMG, Mishra PR (2004). Dual coated erodible microcapsules for modified release of diclofenac sodium. *European Journal of Pharmaceutics and Biopharmaceutics*.

[45] Joseph I, Venkataram S (1995). Indomethacin sustained release from alginate-gelatin or pectin-gelatin coacervates. *International Journal of Pharmaceutics*.

[46] Adeyeye CM, Price JC (1997). Chemical, dissolution stability and microscopic evaluation of suspensions of ibuprofen and sustained release ibuprofen-wax microspheres. *Journal of Microencapsulation*.

[47] Arias JL, López-Viota M, López-Viota J, Delgado ÁV (2009). Development of iron/ethylcellulose (core/shell) nanoparticles loaded with diclofenac sodium for arthritis treatment. *International Journal of Pharmaceutics*.

[48] Conner CS (1984). Oral Gold in arthritis. *Drug Intelligence and Clinical Pharmacy*.

[49] Chen H, Chang X, Du D, Li J, Xu H, Yang X (2006). Microemulsion-based hydrogel formulation of ibuprofen for topical delivery. *International Journal of Pharmaceutics*.

[50] Bodmeier R, Chen H (1990). Indomethacin polymeric nanosuspensions prepared by microfluidization. *Journal of Controlled Release*.

[51] Meyers OL, Klemp P (1981). An oral formulation of gold for the treatment of rheumatoid arthritis. *South African Medical Journal*.

[52] Çomoglu T, Gönül N, Baykara T (2002). The effects of pressure and direct compression on tabletting of microsponges. *International Journal of Pharmaceutics*.

[53] Landewé RBM, Goei Thè HS, van Rijthoven AWAM, Breedveld FC, Dijkmans BAC (1994). A randomized, double-blind, 24-week controlled study of low-dose cyclosporine versus chloroquine for early rheumatoid arthritis. *Arthritis and Rheumatism*.

[54] Harigai T, Hagiwara H, Ogawa Y, Ishizuka T, Kaneda S, Kimura J (2007). Prednisolone phosphate-containing TRX-20 liposomes inhibit cytokine and chemokine production in human fibroblast-like synovial cells: a novel approach to rheumatoid arthritis therapy. *Journal of Pharmacy and Pharmacology*.

[55] Khaled KA, Sarhan HA, Ibrahim MA, Ali AH, Naguib YW (2010). Prednisolone-loaded PLGA microspheres. In vitro characterization and in vivo application in adjuvant-induced arthritis in mice. *American Association of Pharmaceutical Scientists*.

[56] Hwang J, Rodgers K, Oliver JC, Schluep T (2008). *α*-Methylprednisolone conjugated cyclodextrin polymer-based nanoparticles for rheumatoid arthritis therapy. *International Journal of Nanomedicine*.

[57] van Os EC, Zins BJ, Sandborn WJ (1996). Azathioprine pharmacokinetics after intravenous, oral, delayed release oral and rectal foam administration. *Gut*.

[58] Pal R, Chakraborty M, Debnath R, Gupta BK (2009). In vitro-in vivo correlation (IVIVC) study of leflunomide loaded microspheres. *International Journal of Pharmacy and Pharmaceutical Sciences*.

[59] Gupta BK, Pal R, Chakraborty M, Debnath R (2009). Design, evaluation and optimization of microcapsules of leflunomide with Eudragit RL100 and Eudragit RS. 100 by solvent evaporation technique. *Asian Journal of Pharmaceutics*.

[60] Sharma A, Arora S (2012). Formulation and in vitro evaluation of ufasomes for dermal administration of methotrexate. *ISRN Pharmaceutics*.

[61] Prabhu P, Shetty R, Koland M (2012). Investigation of nano lipid vesicles of methotrexate for anti-rheumatoid activity. *International Journal of Nanomedicine*.

[62] Liang LS, Jackson J, Min W, Risovic V, Wasan KM, Burt HM (2004). Methotrexate loaded poly(L-lactic acid) microspheres for intra-articular delivery of methotrexate to the joint. *Journal of Pharmaceutical Sciences*.

[63] Bonetti A, Chatelut E, Kim S (1994). An extended-release formulation of methotrexate for subcutaneous administration. *Cancer Chemotherapy and Pharmacology*.

[64] Patel P, Patel H, Panchal S, Mehta T (2012). Formulation strategies for drug delivery of tacrolimus: an overview. *International Journal of Pharmaceutical Investigation*.

[65] Gabard B, Ellgehausen P (1993). Comparative clinical trial with immediate-release diclofenac pellets, 50 mg TID, and slow-release diclofenac pellets, 75 mg BID. *Current Therapeutic Research*.

[66] Türker S, Erdoğan S, Özer Y, Bilgili H, Deveci S (2008). Enhanced efficacy of diclofenac sodium-loaded lipogelosome formulation in intra-articular treatment of rheumatoid arthritis. *Journal of Drug Targeting*.

[67] Semalty A, Semalty M, Singh D, Rawat MSM (2009). Development and physicochemical evaluation of pharmacosomes of diclofenac. *Acta Pharmaceutica*.

[68] Bhatnagar S, Nakhare S, Vyas SP (1995). Poloxamer-coated three-ply-walled microcapsules for controlled delivery of diclofenac sodium. *Journal of Microencapsulation*.

[69] Tunçay M, Çaliş S, Kaş HS, Ercan MT, Peksoy I, Hincal AA (2000). In vitro and in vivo evaluation of diclofenac sodium loaded albumin microspheres. *Journal of Microencapsulation*.

[70] Setoguchi N, Takamura N, Fujita K (2013). A diclofenac suppository-nabumetone combination therapy for arthritic pain relief and a monitoring method for the diclofenac binding capacity of HSA site II in rheumatoid arthritis. *Biopharmaceutics & Drug Disposition*.

[71] Hu L, Yang J, Liu W, Li L (2011). Preparation and evaluation of ibuprofen-loaded microemulsion for improvement of oral bioavailability. *Drug Delivery*.

[72] Gohel MC, Nagori SA (2010). Fabrication and evaluation of hydrogel thickened microemulsion of ibuprofen for topical delivery. *Indian Journal of Pharmaceutical Education and Research*.

[73] Fernández-Carballido A, Herrero-Vanrell R, Molina-Martínez IT, Pastoriza P (2004). Biodegradable ibuprofen-loaded PLGA microspheres for intraarticular administration: effect of Labrafil addition on release in vitro. *International Journal of Pharmaceutics*.

[74] Irfan M, Verma S, Ram A (2012). Preparation and characterization of ibuprofen loaded transferosome as a novel carrier for transdermal drug delivery system. *Asian Journal of Pharmaceutical and Clinical Research*.

[75] O’Connor TP, Anderson AMR, Lennox B, Muldoon C (1993). A novel sustained-release formulation of ibuprofen provides effective once-daily therapy in the treatment of rheumatoid arthritis and osteoarthritis. *The British Journal of Clinical Practice*.

[76] Kaarela K, Lehtinen K, Makisara P, Holttinen K, Lamminsivu U, Gordin A (1982). Pharmacokinetics and tolerance of slow-release indomethacin tablets in rheumatoid arthritis. *European Journal of Clinical Pharmacology*.

[77] Waller ES (1983). Evaluation of new indomethacin dosage forms. *Pharmacotherapy*.

[78] Chauhan AS, Jain NK, Diwan PV, Khopade AJ (2004). Solubility enhancement of indomethacin with poly(amidoamine) dendrimers and targeting to inflammatory regions of arthritic rats. *Journal of Drug Targeting*.

[79] Srinath P, Vyas SP, Diwan PV (2000). Preparation and pharmacodynamic evaluation of liposomes of indomethacin. *Drug Development and Industrial Pharmacy*.

[80] Soehngen EC, Godin-Ostro E, Fielder FG, Ginsberg RS, Slusher MA, Weiner AL (1988). Encapsulation of indomethacin in liposomes provides protection against both gastric and intestinal ulceration when orally administered to rats. *Arthritis and Rheumatism*.

[81] Bhardwaj P, Chaurasia H, Chaurasia D, Prajapati SK, Singh S (2010). Formulation and in-vitro evaluation of floating microballoons of indomethacin. *Acta Poloniae Pharmaceutica*.

[82] Palakurthi S, Vyas SP, Diwan PV (2005). Biodisposition of PEG-coated lipid microspheres of indomethacin in arthritic rats. *International Journal of Pharmaceutics*.

[83] Shakeel F, Ramadan W, Gargum HM, Singh R (2010). Preparation and in vivo evaluation of indomethacin loaded true nanoemulsions. *Scientia Pharmaceutica*.

[84] Wafin F, Valindas E, Wuolijoki E (1984). Comparison of diclofenac and indomethacin suppositories in rheumatoid arthritis. *Clinical Rheumatology*.

[85] Shinkai N, Korenaga K, Mizu H, Yamauchi H (2008). Intra-articular penetration of ketoprofen and analgesic effects after topical patch application in rats. *Journal of Controlled Release*.

[86] Prajapati CV, Patel RP, Prajapati BG (2012). Formulation, optimization and evaluation of sustained release microsphere of ketoprofen. *Journal of Pharmacy and Bioallied Sciences*.

[87] El Khodairy KA, Eshra AG, Nada AH, Mortada SAM (1992). Preparation and in vitro evaluation of slow release ketoprofen microcapsules formulated into tablets and capsules. *Journal of Microencapsulation*.

[88] Kim BS, Won M, Lee KM, Kim CS (2008). In vitro permeation studies of nanoemulsions containing ketoprofen as a model drug. *Drug Delivery*.

[89] Phillips NC, Thomas DPP, Knight CG, Dingle JT (1979). Liposome-incorporated corticosteroids. II. Therapeutic activity in experimental arthritis. *Annals of the Rheumatic Diseases*.

[90] Davidenkova EF, Ternova NK, Rozenberg OA (1984). Relationship between prolongation of antiinflammatory activity of hydrocortisone incorporated into liposomes and their lipid composition in experimental arthritis. *Bulletin of Experimental Biology and Medicine*.

[91] Metselaar JM, van den Berg WB, Holthuysen AEM, Wauben MHM, Storm G, van Lent PLEM (2004). Liposomal targeting of glucocorticoids to synovial lining cells strongly increases therapeutic benefit in collagen type II arthritis. *Annals of the Rheumatic Diseases*.

[92] Avnir Y, Ulmansky R, Wasserman V (2008). Amphipathic weak acid glucocorticoid prodrugs remote-loaded into sterically stabilized nanoliposomes evaluated in arthritic rats and in a Beagle dog. *Arthritis and Rheumatism*.

[93] Hofkens W, Grevers LC, Walgreen B (2011). Intravenously delivered glucocorticoid liposomes inhibit osteoclast activity and bone erosion in murine antigen-induced arthritis. *Journal of Controlled Release*.

[94] Hofkens W, Storm G, van den Berg WB, van Lent PL (2011). Liposomal targeting of glucocorticoids to the inflamed synovium inhibits cartilage matrix destruction during murine antigen-induced arthritis. *International Journal of Pharmaceutics*.

[95] Ulmansky R, Turjeman K, Baru M (2012). Glucocorticoids in nano-liposomes administered intravenously and subcutaneously to adjuvant arthritis rats are superior to the free drugs in suppressing arthritis and inflammatory cytokines. *Journal of Controlled Release*.

[96] Bonanomi MH, Velvart M, Stimpel M, Roos KM, Fehr K, Weder HG (1987). Studies of pharmacokinetics and therapeutic effects of glucocorticoids entrapped in liposomes after intraarticular application in healthy rabbits and in rabbits with antigen-induced arthritis. *Rheumatology International*.

[97] Bonanomi MH, Velvart M, Weder HG (1987). Fate of different kinds of liposomes containing dexamethasone palmitate after intra-articular injection into rabbit joints. *Journal of Microencapsulation*.

[98] Koning GA, Schiffelers RM, Wauben MHM (2006). Targeting of angiogenic endothelial cells at sites of inflammation by dexamethasone phosphate-containing RGD peptide liposomes inhibits experimental arthritis. *Arthritis and Rheumatism*.

[99] Rauchhaus U, Kinne RW, Pohlers D (2009). Targeted delivery of liposomal dexamethasone phosphate to the spleen provides a persistent therapeutic effect in rat antigen-induced arthritis. *Annals of the Rheumatic Diseases*.

[100] Rauchhaus U, Schwaiger FW, Panzner S (2009). Separating therapeutic efficacy from glucocorticoid side-effects in rodent arthritis using novel, liposomal delivery of dexamethasone phosphate: long-term suppression of arthritis facilitates interval treatment. *Arthritis Research and Therapy*.

[101] Anderson R, Franch A, Castell M (2010). Liposomal encapsulation enhances and prolongs the anti-inflammatory effects of water-soluble dexamethasone phosphate in experimental adjuvant arthritis. *Arthritis Research and Therapy*.

[102] van den Hoven JM, Hofkens W, Wauben MHM (2011). Optimizing the therapeutic index of liposomal glucocorticoids in experimental arthritis. *International Journal of Pharmaceutics*.

[103] Lopez-Garcia F, Vazquez-Auton JM, Gil F (1993). Intra-articular therapy of experimental arthritis with a derivative of triamcinolone acetonide incorporated in liposomes. *The Journal of Pharmacy and Pharmacology*.

[104] Foong WC, Green KL (1988). Retention and distribution of liposome-entrapped [3H]methotrexate injected into normal or arthritic rabbit joints. *Journal of Pharmacy and Pharmacology*.

[105] Foong WC, Green KL (1993). Treatment of antigen-induced arthritis in rabbits with liposome-entrapped methotrexate injected intra-articularly. *Journal of Pharmacy and Pharmacology*.

[106] Williams AS, Camilleri JP, Amos N, Williams BD (1995). Differential effects of methotrexate and liposomally conjugated methotrexate in rat adjuvant-induced arthritis. *Clinical and Experimental Immunology*.

[107] Williams AS, Camilleri JP, Goodfellow RM, Williams BD (1996). A single intra-articular injection of liposomally conjugated methotrexate suppresses joint inflammation in rat antigen-induced arthritis. *British Journal of Rheumatology*.

[108] Williams AS, Jones SG, Goodfellow RM, Amos N, Williams BD (1999). Interleukin-1*β* (IL-1*β*) inhibition: a possible mechanism for the anti-inflammatory potency of liposomally conjugated methotrexate formulations in arthritis. *British Journal of Pharmacology*.

[109] Williams A, Goodfellow R, Topley N, Amos N, Williams B (2000). The suppression of rat collagen-induced arthritis and inhibition of macrophage derived mediator release by liposomal methotrexate formulations. *Inflammation Research*.

[110] Williams AS, Topley N, Dojcinov S, Richards PJ, Williams BD (2001). Amelioration of rat antigen-induced arthritis by liposomally conjugated methotrexate is accompanied by down-regulation of cytokine mRNA expression. *Rheumatology*.

[111] Maria B, Luiza BD, Marie CA, Alexandru BGT, Cristian B (2013). Comparative evaluation of methotrexate toxicity as solution for injection and liposomes following a short term treatment in a murine model of arthritis. Note I. Haematological and biochemical evaluation. *Farmacia*.

[112] van Lent PLEM, van den Bersselaar L, van den Hoek AEM (1993). Reversible depletion of synovial lining cells after intra-articular treatment with liposome-encapsulated dichloromethylene diphosphonate. *Rheumatology International*.

[113] van Lent PLEM, Holthuysen AEM, van Rooijen N, van de Putte LBA, van den Berg WB (1998). Local removal of phagocytic synovial lining cells by clodronate- liposomes decreases cartilage destruction during collagen type II arthritis. *Annals of the Rheumatic Diseases*.

[114] Kinne RW, Schmidt-Weber CB, Hoppe R (1995). Long-term amelioration of rat adjuvant arthritis following systemic elimination of macrophages by clodronate-containing liposomes. *Arthritis and Rheumatism*.

[115] Kinne RW, Schmidt CB, Buchner E, Hoppe R, Nurnberg E, Emmrich F (1995). Treatment of rat arthritides with clodronate-containing liposomes. *Scandinavian Journal of Rheumatology, Supplement*.

[116] Richards PJ, Williams AS, Goodfellow RM, Williams BD (1999). Liposomal clodronate eliminates synovial macrophages, reduces inflammation and ameliorates joint destruction in antigen-induced arthritis. *Rheumatology*.

[117] Barrera P, Blom A, van Lent PL (2000). Synovial macrophage depletion with clodronate-containing liposomes in rheumatoid arthritis. *Arthritis and Rheumatism*.

[118] Ceponis A, Waris E, Monkkonen J (2001). Effects of low-dose, noncytotoxic, intraarticular liposomal clodronate on development of erosions and proteoglycan loss in established antigen-induced arthritis in rabbits. *Arthritis and Rheumatism*.

[119] Richards PJ, Williams BD, Williams AS (2001). Suppression of chronic streptococcal cell wall-induced arthritis in Lewis rats by liposomal clodronate. *Rheumatology*.

[120] Highton J, Guévremont D, Thomson J, Carlisle B, Tucker I (1999). A trial of clodronate-liposomes as anti-macrophage treatment in a sheep model of arthritis. *Clinical and Experimental Rheumatology*.

[121] Corvo ML, Boerman OC, Oyen WJG (1999). Intravenous administration of superoxide dismutase entrapped in long circulating liposomesII. In vivo fate in a rat model of adjuvant arthritis. *Biochimica et Biophysica Acta*.

[122] Corvo ML, Jorge JCS, Van’t Hof R, Cruz MEM, Crommelin DJA, Storm G (2002). Superoxide dismutase entrapped in long-circulating liposomes: formulation design and therapeutic activity in rat adjuvant arthritis. *Biochimica et Biophysica Acta*.

[123] Simões SI, Delgado TC, Lopes RM (2005). Developments in the rat adjuvant arthritis model and its use in therapeutic evaluation of novel non-invasive treatment by SOD in Transfersomes. *Journal of Controlled Release*.

[124] Corvo ML, Boerman OC, Oyen WJG (2000). Subcutaneous administration of superoxide dismutase entrapped in long circulating liposomes: in vivo fate and therapeutic activity in an inflammation model. *Pharmaceutical Research*.

[125] Gaspar MM, Boerman OC, Laverman P, Corvo ML, Storm G, Cruz MEM (2007). Enzymosomes with surface-exposed superoxide dismutase: in vivo behaviour and therapeutic activity in a model of adjuvant arthritis. *Journal of Controlled Release*.

[126] Richard P, Roux H, Mattei JP, Michelson AM, Jadot G (1989). Open clinical study of liposomal superoxide dismutase in the treatment of severe rheumatoid arthritis. *Therapie*.

[127] Trif M, Guillen C, Vaughan DM (2001). Liposomes as possible carriers for lactoferrin in the local treatment of inflammatory diseases. *Experimental Biology and Medicine*.

[128] Trif M, Roseanu A, Brock JH, Brewer JM (2007). Designing lipid nanostructures for local delivery of biologically active macromolecules. *Journal of Liposome Research*.

[129] Watson-Clark RA, Banquerigo ML, Shelly K, Hawthorne MF, Brahn E (1998). Model studies directed toward the application of boron neutron capture therapy to rheumatoid arthritis: boron delivery by liposomes in rat collagen-induced arthritis. *Proceedings of the National Academy of Sciences of the United States of America*.

[130] Gulati M, Grover M, Singh S, Singh M (1998). Lipophilic drug derivatives in liposomes. *International Journal of Pharmaceutics*.

[131] Felnerova D, Viret J-F, Glück R, Moser C (2004). Liposomes and virosomes as delivery systems for antigens, nucleic acids and drugs. *Current Opinion in Biotechnology*.

[132] Mufamadi MS, Pillay V, Choonara YE (2011). A review on composite liposomal technologies for specialized drug delivery. *Journal of Drug Delivery*.

[133] Gangwar M, Singh R, Goel RK, Nath G (2012). Recent advances in various emerging vescicular systems: an overview. *Asian Pacific Journal of Tropical Biomedicine*.

[134] Venkataram S, Awni WM, Jordan K, Rahman YE (1990). Pharmacokinetics of two alternative dosage forms for cyclosporine: liposomes and intralipid. *Journal of Pharmaceutical Sciences*.

[135] Gerwin N, Hops C, Lucke A (2006). Intraarticular drug delivery in osteoarthritis. *Advanced Drug Delivery Reviews*.

[136] Dingle JT, Gordon JL, Hazleman BL (1978). Novel treatment for joint inflammation. *Nature*.

[137] Türker S, Erdoğan S, Özer AY (2005). Scintigraphic imaging of radiolabelled drug delivery systems in rabbits with arthritis. *International Journal of Pharmaceutics*.

[138] Shaw IH, Knight CG, Thomas DP, Phillips NC, Dingle JT (1979). Liposome-incorporated corticosteroids. I. The interaction of liposomal cortical palmitate with inflammatory synovial membrane. *British Journal of Experimental Pathology*.

[139] Harigai T, Hagiwara H, Ogawa Y, Ishizuka T, Kaneda S, Kimura J (2007). Prednisolone phosphate-containing TRX-20 liposomes inhibit cytokine and chemokine production in human fibroblast-like synovial cells: a novel approach to rheumatoid arthritis therapy. *Journal of Pharmacy and Pharmacology*.

[140] Cruz MEM, Gaspar MM, Martins MBF, Corvo ML (2005). Liposomal superoxide dismutases and their use in the treatment of experimental arthritis. *Methods in Enzymology*.

[141] Chang H-I, Yeh M-K (2012). Clinical development of liposome-based drugs: formulation, characterization, and therapeutic efficacy. *International Journal of Nanomedicine*.

[142] Allen TM, Cullis PR (2013). Liposomal drug delivery systems: from concept to clinical applications. *Advance Drug Delivery Reviews*.

